# Lichen planus of uterine cervix - the first report of a novel site of occurrence: a case report

**DOI:** 10.1186/1757-1626-2-9306

**Published:** 2009-12-11

**Authors:** Ruchika Gupta, Bhavna Bansal, Sompal Singh, Indra Yadav, Kusum Gupta, Madhur Kudesia

**Affiliations:** 1 Department of Pathology, All India Institute of Medical Sciences, New Delhi, 110076, India; 2 Department of Pathology, Hindu Rao Hospital, Delhi, 110076, India; 3 Department of Gynecology and Obstetrics, Hindu Rao Hospital, Delhi, 110076, India

## Abstract

**Introduction:**

Lichen planus is an immune mediated inflammatory lesion involving skin and mucosal sites including oral mucosa, vulva and rarely vagina. Lichen planus occurring at mucosal sites has been shown to be associated with squamous cell carcinoma in a proportion of cases. To the best of our knowledge, no case of lichen planus of uterine cervix has been reported in the available literature.

**Case Presentation:**

A 45-year-old female underwent vaginal hysterectomy for uterine prolapse. The resected specimen showed a bluish-colored area in the non-dependent part of the ectocervix. Microscopic sections from this area showed dense lymphocytic infiltrate at the junction of mucosa and submucosa causing disruption of the basal cell layer. On immunohistochemical examination there was predominance of CD8+ T lymphocytes at the junction with scattered CD4+ T lymphocytes, characteristic of lichen planus. Based on the history and negative serum antibody titers, other differential diagnoses including lupus erythematosus and drug reaction were excluded. The patient did not have any cutaneous or oral lesions of lichen planus.

**Conclusion:**

Lichen planus of uterine cervix is a hitherto unreported entity, and is worth studying considering the premalignant potential of lichen planus at other mucosal sites.

## Introduction

Lichen planus is chronic inflammatory mucocutaneous disease with an immunologic etiopathogenesis [1,2]. It most commonly involves skin with rare involvement of oral cavity, nails, vulva and vaginal mucosa [3]. Occurrence of squamous cell carcinoma (SCC) has been reported in oral lichen planus and vulvar lichen planus [4,5]. In oral lichen planus, SCC has been reported in upto 3% of cases [4]. Though uterine cervix is also a mucosal site, no case of lichen planus has been reported in the English literature.

We report the first case of cervical lichen planus with histopathological and immunohistochemical confirmation. Since cervical cancer is one of the commoner cancers in females, report of lichen planus, a pre-neoplastic condition, becomes important. More such cases need to be reported to delineate the biologic significance of the lesion at this site.

## Case presentation

A 45-year-old Indian female presented to the gynecologist with a six-month history of a mass descending down the vagina. She was post menopausal for the last three years and there was no relevant medical or surgical history. Local examination revealed second-degree uterine prolapse with cystocoele and rectocoele. Cervix showed a bluish-colored area measuring 2.5 × 3 cm in size. This area was not related to the most dependent part of cervix. Vulva and vagina were unremarkable. Routine cervical smear was reported as atrophic smear with inflammation. Pre-operative biochemical investigations were unremarkable and she underwent vaginal hysterectomy.

**Figure 1:**
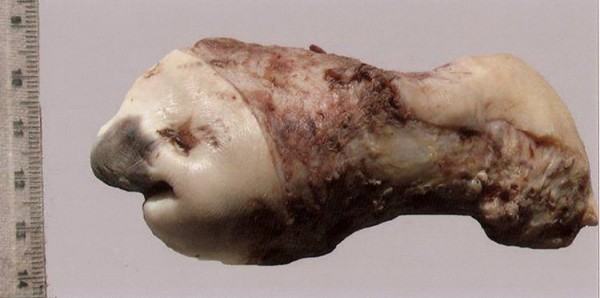
**Gross photograph of the specimen showing a bluish discolored area in the ectocervix**.

**Figure 2:**
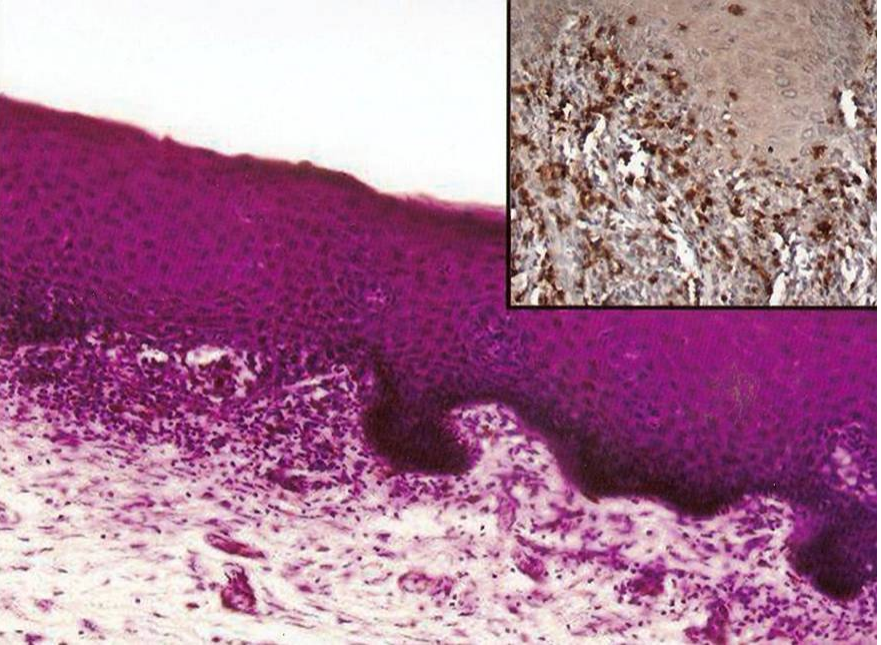
**Photomicrograph demonstrating the lymphocytic infiltrate at the junction of epithelium and subepithelium with destruction of basal layer of the epithelium (H&E×200)**. Inset shows immunostaining for CD8 subset of T-cells with aggregation at the junction and intraepithelial location along with basal layer destruction and exocytosis of lymphocytes into the epithelium (×400).

On gross examination, a bluish discolored area without ulceration measuring 2.5 × 3 × 3 cm in size was seen in the uterine cervix (Figure 1). Endometrial and endocervical cavities and myometrium were unremarkable. Microscopic sections from uterus showed basal endometrium and unremarkable myometrium. The discolored area was processed in entirety and the sections showed mild focal hyperplasia of the epithelium, basal layer destruction and a dense band-like lymphocytic infiltrate at the junction of epithelium and the subepithelium with exocytosis of lymphocytes in the epithelium (Figure 2). There was no evidence of dysplasia in the lining epithelium. No appreciable congestion was seen and rest of the cervix showed only minimal chronic inflammatory infiltrate in the subepithelium. Immunohistochemistry was performed for CD4 and CD8 subsets of T cells (Biogenex, San Ramon, USA) using peroxidase as the enzyme. Clusters of CD8 + T cells were seen at the junction of mucosa and submucosa, specifically in areas of basement membrane disruption. CD8+ T cells were also seen infiltrating the lower parts of epidermis (Figure 2). The CD4+ cells were seen scattered with no preferential localization. Based on the histological features, a diagnosis of an inflammatory lesion with interface involvement was considered and the various causes looked for. Systemic lupus erythematosus was excluded on the basis of absence of clinical features and negative serum antibody titers (anti-nuclear factor and anti-dsDNA). Extensive history-taking did not reveal any drug intake. On extensive examination of the patient, no evidence of LP at other cutaneous or mucosal sites was found. Thus a final pathologic diagnosis of isolated lichen planus of the uterine cervix was rendered.

## Discussion

Lichen planus is a chronic immune-mediated mucocutaneous disease with characteristic violaceous polygonal flat-topped papules and plaques. It occurs mainly on flexor surfaces of extremities and trunk, though can also involve scalp, nails, oral and genital mucosa [3]. Genital lichen planus is seen typically on the vulva, and rarely on the vagina, as part of the vulvo-vaginal-gingival syndrome [6]. In contrast to lesions of cutaneous LP, mucosal lesions, including oral cavity, do not show compact orthokeratosis or hypergranulosis. Instead, parakeratosis with frequently atrophic epithelium is seen, sometimes with ulceration of the epithelium [7]. Till date no case of lichen planus involving uterine cervix has been reported in the available literature. The lesion in our case was seen as an isolated finding, in the absence of cutaneous lesions. The same is true in a proportion of oral lesions which occur without cutaneous involvement.

The pathogenesis of lichen planus is not certain, though many studies have supported an immunologic mechanism. The role of T lymphocytes has been emphasized with cytotoxic activity of the CD8+ve subset of T lymphocytes responsible for keratinocyte damage [1,2]. In our case, immunohistochemistry for T cell subsets showed specific localization of CD8-positive cells at the junction of mucosa and submucosa associated with basement membrane disruption, supporting the role of CD8-positive T cells in the pathogenesis of this entity. The etiology of LP in oral cavity has been investigated extensively. Various infectious agents, including bacterial, fungal (Candida) and viral organisms have been implicated as etiologic agents of oral LP. Among viruses, human papilloma virus (HPV) has also been found in oral LP [8]. Since HPV infection is common in cervix, its role in causation of LP of cervix needs to be evaluated after more cases of cervical LP are added in the literature. In our patient, staining for HPV could not be performed. However, we propose that uterine prolapse in our patient may have exposed the cervix to the environmental agents leading to the development of lichen planus.

Malignant transformation of cutaneous lesions of lichen planus has been reported in less than 1% of cases [9]. Similar phenomenon has been documented in 0.3-3% of cases of oral lesions [4,10]. Rare cases of squamous cell carcinoma have been reported in vulvar lichen planus [5]. Cervix is another mucosal site, where we report the first case of lichen planus, its pre-malignant potential remains to be seen.

The gross appearance of cervical lesion needs to be differentiated from congestion due to prolapse of uterus. Congestion occurs as bluish-brown discoloration on the dependent portion of the cervix whereas lichen planus has no such preference. Histologic examination differentiates between congestion and lichen planus, since congestion does not exhibit the band-like infiltrate observed in lichen planus with destruction of the overlying epithelium. Characteristic localization of T-cell subsets using Immunohistochemistry for CD4 and CD8 further resolves the matter. Other lesions, like involvement in systemic lupus erythematosus and drug reactions were excluded with the history and negative serologic findings.

## Conclusion

Cervical lichen planus is a hitherto unreported entity. We report the first case of lichen planus of uterine cervix confirmed by histopathology and immunohistochemistry. Since squamous cell carcinoma has been reported in long-standing lesions of mucosal lichen planus, existence of lichen planus in uterine cervix needs to be identified and more cases must be added to the literature in order to assist in the further study on this subject.

## Consent

Written consent was obtained from the patient for publication of this case report and accompanying images. A copy of the written consent is available for review from the journal's Editor-in-Chief.

## Competing interests

The authors declare that they have no competing interests.

## Authors' contributions

RG was involved in drafting the manuscript and conducting a literature search. BB and SS were involved in signing out the case on histopathology and helped in literature review. IY was the gynecologist in-charge of the day-to-day care of the patient. KG helped in signing out of the case and finalizing the manuscript. MK assisted in critical evaluation of the manuscript and approved its final form.
